# Multifocal hepatoblastoma in a 6-month-old girl with trisomy 18: a case report

**DOI:** 10.4076/1752-1947-3-8319

**Published:** 2009-06-23

**Authors:** Lidija Kitanovski, Zdenka Ovcak, Janez Jazbec

**Affiliations:** 1 University Medical Centre Ljubljana, Department of Pediatrics, Hematooncology Division, Vrazov trg 1, 1000 Ljubljana, Slovenia; 2 Institute of Pathology, Medical Faculty, University of Ljubljana, Korytkova 2, 1000 Ljubljana, Slovenia

## Abstract

**Introduction:**

Edward's syndrome (trisomy 18) is a rare entity with a reported incidence of 1/3000 to 1/7000 births. Less than 10% of patients survive beyond the first year of life, which may influence the fact that malignant tumors are rarely reported in association with this syndrome.

**Case presentation:**

The authors report a rare case of a 6-month-old girl with trisomy 18 and multifocal hepatoblastoma. The course of the disease, autopsy results and review of the literature are presented.

**Conclusion:**

Our case represents the seventh published case of hepatoblastoma in a patient with trisomy 18. All of the seven published cases were women, possibly due to the high preponderance of females among the children with Edward's syndrome and longer survival of females with trisomy 18 compared to males. Since both trisomy 18 and hepatoblastoma are rare conditions, the probability that a child with trisomy 18 will independently develop a hepatoblastoma is very low. Therefore, we believe that the existence of these cases in children with trisomy 18 indicates a significant association. It can be assumed that trisomy 18 potentiates the development of hepatoblastoma. Careful clinical and post-mortem studies are needed to recognize the real frequency of hepatoblastoma in children with trisomy 18, who might die from different causes with unrecognizable hepatoblastoma.

## Introduction

Edward's syndrome (ES) was first recognized as a specific entity in 1960 by the discovery of the extra 18 chromosome in babies with a particular pattern of malformations [1]. Children with trisomy 18 may have intrauterine growth retardation, microcephaly, short stature, mental retardation, cranio-facial abnormalities such as a small face, prominent occiput, short palpebral fissures, small mouth; limb abnormalities including overlapping fingers, camptodactyly and nail hypoplasia; congenital heart disease, omphalocele, horseshoe kidney, hypertonia, and short sternum. It has been reported that the incidence of ES is 1 in 3000 to 7000 births [2,3] and that survival beyond infancy is unusual [2,4]. Less than 10% survive the first year [2,4]. There is a 3:1 preponderance of females to males [5]. Malignant tumors are infrequently reported in ES, perhaps due to high early mortality.

Hepatoblastoma (HB) sometimes occurs in patients with congenital malformations [6], particularly in Beckwith-Wiedemann syndrome. It is a rare tumor of infancy and childhood with an annual incidence rate of approximately 1.8 per million in children less than 15 years of age [7]. The majority of HB are diagnosed before age two in otherwise normal children and there is a 1.4:1 to 2:1 predominance in males [6].

We present a child with ES who developed HB at the age of 6 months.

## Case presentation

A female, Caucasian, newborn girl was born to a 28-year-old mother after her first pregnancy at 38 weeks of gestation. The pregnancy was uneventful, except for colpitis, until the 29th gestational week when intrauterine growth retardation was noticed. The infant birth weight was 1630 g, birth length was 43 cm, and head circumference was 32.5 cm. The family history was unremarkable. The child had prominent occiput, micrognathia, high palate, low-set ears, overlapping fingers of both hands, bilateral preauricular adnexes and a red pedunculated tumor on the left cheek, diagnosed as a hamartoma. Hypotonia, absent swallowing reflex and abnormal spontaneous movements were observed. Chromosome analysis of the peripheral blood cells revealed 47,XX,+18 chromosome. Echocardiography and abdominal ultrasound examination were normal, while the ultrasound of the head revealed agenesis of the corpus callosum. Due to respiratory failure after birth, she was artificially ventilated for 2 weeks. Thereafter she was nursed at home, nourished through a gastrointestinal tube and her clinical course was uneventful. At the age of 6 months, after she had been treated for a urinary tract infection, hepatomegaly was noticed. Abdominal ultrasound revealed three well-defined hepatic masses. The largest one was 8.3 × 5.6 × 9.6 cm in size, and the two smaller masses were approximately 3.2 and 3.7 cm in size. Only a minority of the liver parenchyma appeared normal. Hepatoblastoma was confirmed by fine needle aspiration biopsy and an increased level of α-fetoprotein (51542 IU/mL). Pulmonary X-ray was normal. The infant was not treated for the tumor in accordance with the parents' decision. She was nursed at home and only analgesic drugs were given.

One month later, she was admitted to hospital due to restlessness, vomiting and cough for the previous 4 days. She was in pain, febrile, icteric and protected her left arm. Hypercalcemia (calcium 4.5 mmol/L) and fracture of the left humerus were observed. She was treated with intravenous bisphosphonates, analgesics and the left arm was immobilized. In the following hours, she became progressively dyspnoic and died on the next day.

**Figure 1:**
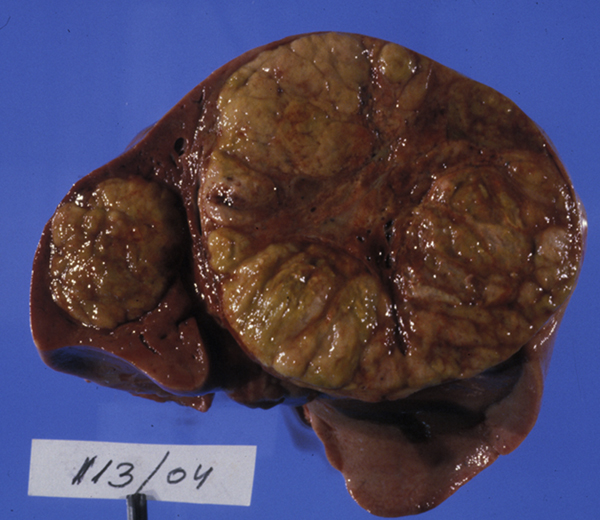
**A multicentric lobulated liver tumor involves almost the entire parenchyma**. Two tumor nodules clearly separated from each other are visible in the cross-section of the liver at autopsy.

**Figure 2:**
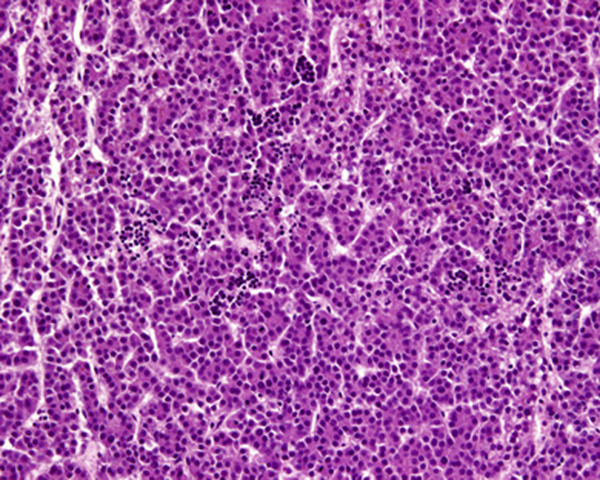
**Fetal type tumor cells resembling hepatocytes are arranged in trabeculae and plates**. Foci of extramedullary hematopoiesis are also present. Hematoxylin and eosin stain, ×40.

At autopsy, the liver (741 g) was almost completely overgrown with a multicentric tumor. The largest mass measured 10 × 9 × 8 cm (Figure 1). The histopathologic diagnosis was epithelial HB - fetal type with typical histologic appearance (Figure 2). In the field of the humerus fracture, no tumorous tissue was found on microscopic examination. Disseminated microscopic intravascular coagulation was observed in the lungs and kidneys. Neuropathologic autopsy revealed polymicrogyria, atrophy of the cerebellum and white matter, hypoplasia of the corpus callosum, dysplasia of the hippocampus, atrophic pontocerebellar connections, dysplasia of the lower olivary nucleus typical of trisomy 18 and atrophic pyramids in the medulla oblongata. No additional abnormalities of the heart, lungs, kidneys, suprarenal glands and gut were found.

## Discussion

Neoplasias are uncommon in ES, possibly due to the high mortality in the first year of life. Nevertheless, there are reports of one neurogenic tumor [8] and at least six Wilms tumors, in children with ES [9]-[12].

Congenital abnormalities have also been recognized in patients with HB [11]-[13] which, after Wilms tumor, is the second most common tumor associated with congenital anomalies.

Hepatoblastoma has been documented to be related to Beckwith-Wiedemann syndrome, hemi-hypertrophy [13] and Prader-Willi syndrome [14]. Moreover, six cases of HB in children with trisomy 18 have been published since 1987, when Dasouki and Barr reported the first case, which was presumed to be HB [15]-[20]. The last review of published cases was carried out by Maruyama *et al.*[20]. All of the cases were girls, and half of the cases were older than 1 year at the time of recognition of HB. Five of them had karyotype 47,XX,+18 [15]-[17,19,20], and in another one [18], chromosomal analysis of peripheral blood culture showed mosaic trisomy 47,XX,+18/46,XX (5:1). There is another case mentioned by Bove *et al.*[15]. It can be concluded that it refers to a 1-year-old boy, mosaic for trisomy 18. Gut abnormalities were present in three of the patients (malrotation of the gut in all three, ectopic pancreas in two, omphalocele in one) [15,17,20], while morphological abnormalities of the liver had not been observed, except for a deep cleft between the hepatic lobes in one patient [15]. Apart for neurologic abnormalities, no visceral irregularities were found in our patient. In three of the cases, where the tumors were cytogenetically analyzed, excessive chromosome 18 was found in the tumor tissue [15,18,19]. Epithelial type HB with different histological patterns was diagnosed in all patients [15,17]-[20].

The liver tumors were resected in three cases; two patients were alive with no evidence of recurrence at 3 and 4 years of age [18,19], the other died due to widespread bone metastases [15]. Among the untreated patients, HB was an incidental finding at autopsy in one of two patients who died from cardiac failure [17,20], while our patient and the one with presumed HB [16] died due to progression of malignant disease.

## Conclusion

Our case represents the seventh published case of HB in trisomy 18 and, together with the unpublished case mentioned by Bove *et al.*[15], represents the eighth known case of HB in children with trisomy 18. All of the seven published cases were females, possibly due to the high preponderance of females among the children with ES and longer survival of females with trisomy 18 compared to males [4]. Since both trisomy 18 and HB are rare conditions, the probability that a child with trisomy 18 will independently develop a HB is very low. Therefore, we believe that the existence of these cases in children with trisomy 18 indicates a significant association. It can be assumed that trisomy 18 potentiates the development of HB. Careful clinical and post-mortem studies are required to recognize the real frequency of HB in children with trisomy 18, who might die from different causes with unrecognizable HB.

## Abbreviations

HB: Hepatoblastoma; Trisomy 18: Edward's syndrome.

## Consent

Written informed consent was obtained from the parents for publication of this case report and any accompanying images. A copy of the written consent is available for review by the Editor-in-Chief of this journal.

## Competing interests

The authors declare that they have no competing interests.

## Authors' contributions

LK and JJ were treating physicians and wrote the manuscript. ZO did the autopsy and described autopsy results, pathological description and did the figures.
